# PLGA Core-Shell Nano/Microparticle Delivery System for Biomedical Application

**DOI:** 10.3390/polym13203471

**Published:** 2021-10-10

**Authors:** Se Min Kim, Madhumita Patel, Rajkumar Patel

**Affiliations:** 1 Life Science and Biotechnology Department (LSBT), Underwood Division (UD), Underwood International College, Yonsei University, Sinchon, Seoul 03722, Korea; minisemin@yonsei.ac.kr; 2 Department of Chemistry and Nanoscience, Ewha Woman’s University, 52 Ewhayeodae-gil, Seodaemun-gu, Seoul 03760, Korea; madhurk29@gmail.com; 3 Energy and Environmental Science and Engineering (EESE), Integrated Science and Engineering Division (ISED), Underwood International College, Yonsei University, 85 Songdogwahak-ro, Yeonsugu, Incheon 21983, Korea

**Keywords:** drug delivery, core–shell particle, tissue engineering, periodontal regeneration, anti-cancer activity

## Abstract

Core–shell particles are very well known for their unique features. Their distinctive inner core and outer shell structure allowed promising biomedical applications at both nanometer and micrometer scales. The primary role of core–shell particles is to deliver the loaded drugs as they are capable of sequence-controlled release and provide protection of drugs. Among other biomedical polymers, poly (lactic-co-glycolic acid) (PLGA), a food and drug administration (FDA)-approved polymer, has been recognized for the vehicle material. This review introduces PLGA core–shell nano/microparticles and summarizes various drug-delivery systems based on these particles for cancer therapy and tissue regeneration. Tissue regeneration mainly includes bone, cartilage, and periodontal regeneration.

## 1. Introduction

A drug-delivery system (DDS) based on nano/microparticles has gained attention during the last few decades since these nano/micro vehicles protect loaded drugs, serve as a localized scaffold, and control its release kinetics. These features allow us to have the same treatment effect with less drug dosage, thus reducing possible side effects [1]. What makes nano/microparticle-based DDS even more attractive is the fact that these particles can be modified to have various abilities, like targeting, multiple drug delivery, or being responsive to light, pH, temperature, magnetic field, or different extracellular environments [2,3].

Drug carriers should be compatible with living tissue and degrade into non-toxic organic compounds. A widely used drug carrier is poly(lactic-co-glycolic acid) (PLGA), which is also approved by the U.S. Food and Drug Administration (FDA), thanks to its excellent biodegradable and biocompatible properties [4]. Along with PLGA, poly(lactic acid) (PLA), poly(vinyl alcohol) (PVA), poly(L-lactic acid) (PLLA), poly(ethylene glycol) (PEG), poly(glycolic acid) (PGA), and chitosan (CS) have also been frequently used since they have good biodegradability [5]. The common problem of utilizing PLGA arises when encapsulating hydrophilic agents, since PLGA is a highly hydrophobic polymer. Also, to prepare PLGA, toxic organic solvents are usually used, and these solvents remain in the particle [6].

Core–shell microspheres have two or more distinct layers made of the same or different materials which can each be loaded with bioactive molecules [7]. Depending on the purpose, these core–shell particles have varying core shapes, internal structures, shell thicknesses, and surface morphologies. These factors are the key determinants of loading efficiency and release kinetics [8]. The size of the microspheres usually ranges between 10–200 μm because too small microspheres (< 10 μm) will get engulfed by immune cells and too big microspheres (> 200 μm) will cause inflammation [8]. Numerous techniques for synthesizing core–shell microspheres are developed, including polymerization, spray drying, solvent evaporation, and self-assembly [9]. Using microfluidic devices allows fabricating microspheres with uniform size and thickness, and also has the advantages of higher encapsulation efficiency for both hydrophilic and hydrophobic agents [10].

Cancer therapy using PLGA microspheres includes chemotherapy, photothermal therapy, hormonal therapy, immunotherapy, and others. Like other applications, two or more cancer treatment methods are frequently combined to seek synergistic effects [11,12,13,14,15]. Microspheres are often targeted to cancer cells by adding receptors to the outermost layer or by making them respond to cancer-specific extracellular environments [12]. Core–shell microparticles are also applied to tissue regeneration, as an alternative solution for transplanting and autologous cell therapy. Some application examples include bone regeneration, cartilage, and periodontal regeneration. Microspheres up to 100 μm are said to be suitable for these cases [16]. Strategy that sequentially releases growth factor and differentiation factor is commonly used [8,17,18,19,20,21]. As per our knowledge, several review literatures have been published related to PLGA microspheres for biomedical applications [22,23,24,25]. However, a smaller number of reviews have been reported on the PLGA core–shell microsphere [26,27]. In this review, we summarized the current state of core–shell nano/micro PLGA particles for drug delivery, cancer therapy, and tissue regeneration. In the tissue regeneration section, we especially include bone, cartilage, and periodontal regeneration.

## 2. Drug Delivery

The nano/microparticle-based DDS process can be summarized into two steps: targeting the drug delivery site and releasing the encapsulated agent at the desired time and rate [28]. Targeting can be done by direct injection, receptor attachment, or the addition of nanoparticles that respond to certain bioenvironments. The performance of the designed DDS is commonly assessed by their drug encapsulation efficiency, loading capacity, release pattern, degradation, and other factors. These characteristics are highly dependent on the microsphere’s shape, size, surface, and thickness [8]. The main challenges of using PLGA microspheres are reducing burst release and increasing encapsulation efficiency of hydrophilic drugs. To overcome this issue, additional functional layers, like hydrophilic gels, are included to the PLGA core–shell microspheres, and methods like coaxial electrohydrodynamic atomization (CHEDA) are practiced [29,30,31]. The core–shell particles have been used more frequently in drug delivery owing to the synergistic effect. 

**Figure 1:**
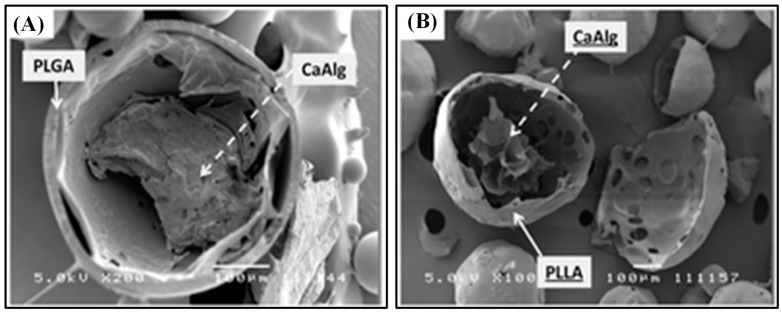
SEM image of a cross-sectioned (**A**) alginate–PLGA particle, and (**B**) alginate–PLLA particle. The scale bar is 100 μm. Reproduced with permission from [32].

Recently, Yu et al. designed PLGA microspheres with gel cores to obtain high encapsulation efficiency for small and highly soluble drugs. For the inner phase, water, 5% gelatin, 25% Pluronic 407 (F127), and losartan potassium (LP) powder were used. The in vitro study revealed that microcapsules with water core had a short release period (up to 16 d) while 5% gelatin core showed a stable release profile (up to 30d), which makes it more suitable for sustained drug release. The LP powder group demonstrated a slow release rate at first, but increased burst release was observed over time and the peak concentration was reached at day 14. The F127 core group showed very slow release at first, but as F127 swelled and cracked the microcapsules, the drugs were rapidly released, which makes them appropriate for delayed release [30]. Similarly, composite hydrogel–PLGA particulate systems are designed to have a hydrogel core which efficiently loads hydrophilic drugs and a polyester shell which limits the rate of drug diffusion via limiting the water influx rate. Fabrication of such a system is time-consuming and requires multiple steps. Thus, a single-step fabrication technique was designed, which is called concurrent ionotropic gelation and solvent extraction. Using this technique, hydrophobic shells with a gel core were produced, such as alginate–PLGA microparticles (Alg–PLGA MP) and alginate–PLLA microparticles (Alg–PLLA MP) (Figure 1). Metoclopramide hydrochloride (MCA) was loaded in these systems as a model drug, and the encapsulation efficiencies were 59.96 ± 13.82% and 66.52 ± 2.23% each. Without an alginate core, they were 30.1 ± 2.40% and 32.26 ± 1.99% each. Also, Alg–PLLA and Alg–PLGA MP microparticles showed prolonged release patterns up to 7 and 4 d, respectively, while naked beads demonstrated total burst release within 1 d [32].

The Poloxamer hydrogel core also increased the encapsulation efficiency of a small, hydrophilic, cationic, and acid-stable peptide. The core–shell PLGA microspheres encapsulated in Goserelin was synthesized by double-emulsion–solvent evaporation method. Poloxamer hydrogel has a disperse phase that enables regulating the release pattern by manipulating pH. The microspheres showed a Di-Depot structure which means that Goserelin acetate (GOS) was scattered throughout PLGA (PLGA depot) and also encapsulated inside the hydrogel core (hydrogel depot). The Di-Depot structure allowed high encapsulation efficiency of 94.16%, and resulted in the release pattern of two peaks, where GOS in PLGA depots diffused first followed by the diffusion of GOS in Poloxamer hydrogel depots. The delay between two release stages could be minimized by decreasing the inner phase pH, since acetic acid provoked the heterogeneous degradation [33]. Another aqueous core–PLGA microcapsule also illustrated a 2.5-fold increase in encapsulation efficiency (31.6%) in comparison to the PLGA microspheres (12.7%). The microcapsules were synthesized by a new method—acetone–water in oil emulsion followed by internal phase separation. The resulting aqueous core–PLGA microcapsules were spherical, poly-nuclear, and their average size was 1.1 ± 0.39 μm. They were able to encapsulate risedronate sodium, a water-soluble model drug, and the microcapsules exhibited a sustained release behavior that followed the diffusion-controlled Higuchi model [34]. Similarly, one more hydrophilic model drug, fluorescein sodium, was encapsulated in the aqueous core of PLGA and PLA microshell. The internal phase separation technique was used and the internal phase polymer to water ratio for maximizing the encapsulation efficiency was 1 to 3. The synthesized PLGA and PLA microvehicles exhibited sustained release over 7 and 49 d, respectively [35]. However, the hydrophobic nature of PLGA often requires using harmful organic solvents to make aqueous core microcapsules. Therefore, Nomura et al. attempted to solve this problem by utilizing a plant oil for the continuous phase in a single emulsion method. It achieved the encapsulation efficiency of Rhodamine B up to 95.8% [6].

**Figure 2:**
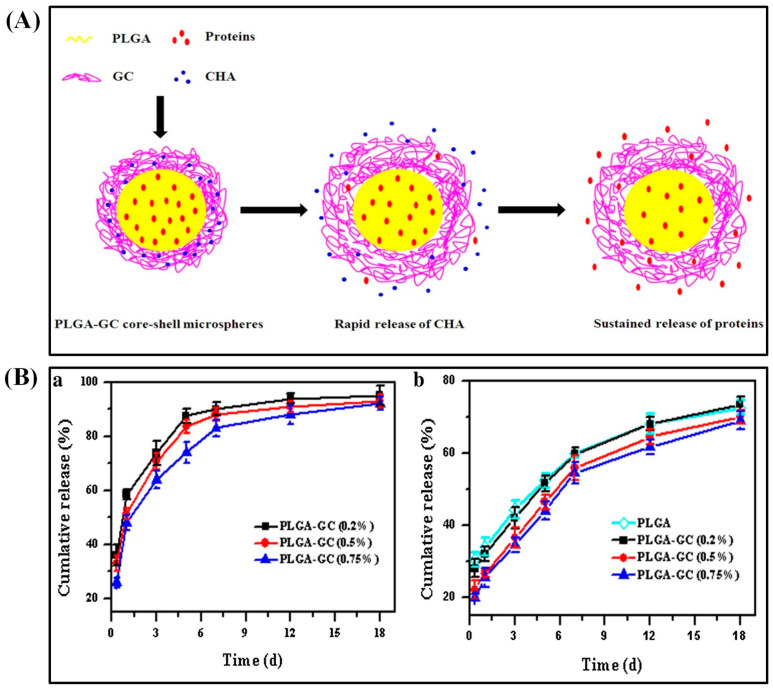
Schematic diagram of the co-delivery mechanism of CHA and proteins from the PLGA–GC core–shell microvehicles (**A**). In vitro release profiles of CHA and BSA from PLGA–GC microspheres (**B**). Reproduced with permission from [37].

In an alternative way, loading biomolecules in the PLGA core successfully suppressed the initial burst. For instance, PLGA double-walled microspheres reduced the initial burst of etanidazole when encapsulated in the core [36]. Chen et al. achieved the sequential release of antibacterial agents and growth factors to heal chronic wounds. PLGA–glycol chitosan (GC) core–shell microspheres were fabricated by w/o/w emulsion–solvent evaporation method and loaded with basic fibroblast growth factor (bFGF) proteins in the PLGA core (Figure 2A). Additionally, chlorhexidine acetate (CHA), an antibacterial agent, was incorporated into the GC shell. The GC coating on the shell increased the average size of PLGA–GC microparticles (5.71–5.88 μm). CHA was rapidly released while bFGF was released sustainably. The loading content of BSA and CHA was 4.41–4.44% (74.3–76.1% encapsulation efficiency) and 1.42–1.78%, respectively. After 1 w, 83–90% of loaded CHA was freed, whereas 69–73% of BSA was released from the core within 18 d (Figure 2B). Positively charged GC allowed easier interaction between microspheres and negatively charged cell membranes by ionic adsorption. These microspheres were proved to be biocompatible with 3T3 fibroblast cells in vitro [37]. Chitosan shell on PLGA microparticles controlled the burst release as well as acidic degradation. These microparticles were synthesized by re-emulsification method and ranged between 32.3 and 45.2 μm size. These particles with multiple PLGA cores loaded with glial cell line-derived neurotrophic factor (GDNF) for neuron regeneration. In vitro study indicated that sustained release as well as neutralization of the acid products were achieved by this multi-core PLGA–chitosan shell microparticles [38]. Likewise, the PLGA core with alginate shell reduced the initial burst and increased the drug-release profiles. The model drug of rifampicin encapsulated in the PLGA core exhibited a sigmoid release pattern. The microsphere is prepared by capillary microfluidic method with uniform size (ranging from 15 to 50 μm) where the release profile depended on the size and the shape of their carriers. The shell could enhance the encapsulation efficiency, play a role as a buffer, and achieve almost zero-order release pattern [39].

Along with microparticles, nano-sized particles, or nanoparticles (NPs) are frequently applied to DDS as they are also capable to deliver appropriate amount of drug to the targeted site at desired time [40]. Recently, several researchers found outstanding results for core/shell nanoparticles in drug delivery. Core–shell nanoparticles secure their loadings from biodegrading agents, and their surface is modified to interact with targeted cells by providing a binding site to the receptors. For example, a nanocomposite QRu-PLGA–RES–DS–NPs, was synthesized to effectively encapsulate and deliver resveratrol (RES), a polyphenolic drug, for Rheumatoid arthritis (RA) treatment. RES can inhibit the inflammation of RA by inducing M1-type macrophages to transform into M2-type macrophages. The quadrilateral ruthenium nanoparticles (QRuNPs) in the core generates heat when exposed to light, and the dextran sulfate (DS) turned PLGA shell to be thermosensitive. One more function of DS is targeting the macrophages by binding to their scavenger receptors. The water solubility of RES was improved by QRu–PLGA–DS NPs from 0.03 mg/mL to 0.11 mg/mL. PLGA’s encapsulation efficiency of RES was 10.2% and RES was rapidly released by the photothermal activity of QRu NPs. In vivo study demonstrated the reversed number of M1 and M2 macrophages in paws and restricted M1 macrophages from recruiting and infiltrating, effectively improving the inflammatory micro environment [41]. In another study, for mycobacterium tuberculosis (MTB) treatment, PLGA–PEG–PLGA tri-block copolymers were designed to stabilize and uniformly distribute the toxic drug isoniazid (INH) (Pyridine 4-carboxylic acid hydrazide). INH was loaded in three parts: on the surface, in the interstitial space, and inside the individual nanoparticles. Thus, the NPs’ loading efficiency (12.8%–18.67%) and the drug content (6.4-8.9%) were high. In vitro release study revealed an initial burst from the surface followed by uniform release which lasted longer from the interstitial space and inside the individual NPs [42].

Reduction of systemic clearance, prolonged circulation, half-life in vivo with high encapsulation efficiency is crucial for the successful DDS. In a core–shell nanoparticle, steric stabilization and modifiable surface are provided by a hydrophilic shell. Chan et al. designed a combined drug-delivery system of liposomal and polymeric nanoparticles which demonstrated both polymers’ combined advantages for controlled drug delivery. PEG core–shell NPs were prepared via self-assembly combined with the modified version of nanoprecipitation. The resulting nanoparticles contained a PLGA (hydrophobic) core, a PEG (hydrophilic) shell, and a soybean lecithin monolayer between them. PLGA allowed the encapsulation of high amounts of hydrophobic drugs, and the PEG shell provided electrostatic and steric stabilizations. The average diameter was from 60 nm to 70 nm while the zeta potential was from 40 mV to −60 mV. Docetaxel, a model chemotherapy drug, loaded in the PLGA–lecithin–PEG core–shell NPs achieved a maximum encapsulation efficiency of 62% and the releasing rate was controlled by the lipid concentration. Lipid monolayer acted as a molecular fence that prevented PLGA polymers from hydrolysis and erosion from H_2_O [43]. 

Co-delivery of two drugs using a single nanocarrier achieved synchronized pharmacokinetics for both drugs. Yeh et al. loaded two drugs Budesonide (hydrophobic) and Theophylline (hydrophilic) in the PLGA core, resulted in more than 95% drug encapsulation efficiency while executing sustained release. Dual-capillary electrospray-dry (ES) method allowed the fabrication of uniformly sized nanoparticles. By increasing shell thickness and using low-molecular-weight PLGA polymer with low LA/GA ratio, sustained release of Theophylline was accomplished. In a case where Budesonide and Theophylline were both encapsulated inside the core, the release rate was the lowest. This is because if different agents are encapsulated in different layers, then empty spaces are created after releasing the shell-loaded agent, which increases the infusion of release medium [10]. Similarly, the prolonged releasing of Gemcitabine, a highly hydrophilic drug, was controlled by bovine serum albumin (BSA)–PLGA core–shell nanoparticles. Hydrophilic BSA core enhanced encapsulation, and hydrophobic PLGA shell controlled the drug release from the system. The optimal size of the particles was 243 nm, with 40.5% encapsulation efficiency and 8.8% *w*/*w* drug loading. In vitro study observed the sustained release pattern that lasted 12 h, and cellular uptake studies showed a quick uptake within 2 h [44].

Recently, magnetic nanoparticles (MNPs) are gaining attention among the other inorganic particles for nano vehicles. MNP’s superparamagnetic feature holds the medicine in the targeted area and applying an external magnetic field can control release. Amphotericin B (AmB), an antibiotic used against fungal infections, encapsulated in MNPs with three different shell types: PVP–PEG (100:20, *w*/*w*), PLGA–PEG (100:20, *w*/*w*), and PLGA–PEG (50:10, *w*/*w*). PLGA–PEG (100:20) nanoparticles demonstrated the best controlled release rate as PLGAs’ high molecular weight and low solubility prolonged the release of AmB. Also, PLGA–PEG nanoparticles (both 100:20 ratio and 50:10 ratio) indicated high AmB activity against *Candida albicans* [45]. The (±)-α-Tocopherol (TP), one type of vitamin E, encapsulated in different nanoparticles: PLA, PLGA65, and PLGA75 where PLA nanoparticles achieved 14.7% drug loading, in which 65% of the TP molecules were loaded in its core and the rest remained on the shell surface. Both PLGA-based nanoparticles showed increased drug loading (PLGA65: 18%, PLGA75: 16%) and higher encapsulation efficiency (PLA: 69.1%, PLGA65: 87.7%, and PLGA75: 75.7%) while TP molecules were more concentrated in the core. The drug-release profile showed fast release for 30 mins, and slow dissolution afterwards. The less the lactide part, the slower the active substance was released (PLA, 35.0%, PLGA75, 28.3%, and PLGA65, 19.8%) in 7 h [46].

Although the core–shell nanoparticles have great potential for drug delivery, preserving them for a long time is challenging because lyophilization leads to the aggregation of nanoparticles. As one solution, PLA and PLGA–core–poly (N-isopropyl acrylamide) (pNIPAM) – shell microparticles were prepared by radical precipitation polymerization subsequent to a single emulsion technique. The pNIPAM shell exhibited an enhanced ability to release the antihypertensive drug ramipril and prevented clustering of PLA and PLGA cores. The core–shell PLA/PLGA–pNIPAM particles had an average size of 157 nm (PLA) and 105 nm (PLGA) each. The total drug content and encapsulation efficiency of PLGA core-pNIPAM shell was 91% and 78% each, and PLA core–pNIPAM shell were 73% and 60% each. It turned out that both particles with a pNIPAM shell had higher encapsulation efficiency than the conventional PLGA core (52.4%) and PLA core (47.42%). In addition, within 24 h, the PLGA–pNIPAM core–shell microparticles demonstrated a sustained releasing pattern of 86% of the model drug [47].

### Anti-Cancer Drug Delivery

Despite long years of therapy investigation, cancer is still a major cause of death [48]. The most common cancer therapies include surgical removal, radiotherapy, and chemotherapy. The major challenges of chemotherapy are localizing drug injection sites to reduce the cytotoxic effect of drugs to non-cancer cells and minimizing the development of drug resistance [49]. Nano drug carriers are advantageous in this aspect; however, problems still arise for their clinical applications. Current generations of nanovehicles are known to lack efficiency, since only 0.7% of the injected nanoparticles actually reach their targeted tumor cells [50]. This is because multiple steps are involved in drug delivery, and each step presents unique difficulties. When nanocarriers are injected into the patient, they initially follow blood circulation and penetrate the tumor tissue. Desirably, nanoparticles are then engulfed into those tumor cells by endocytosis and released in intracellular regions, like the nucleus [51]. This means that the nanoparticles are required to efficiently penetrate the endothelial barriers, interstitial tumor matrix, and cellular barriers, only release their loadings at the final step, and avoid undesirable interactions with untargeted cells—especially the immune cells. Small nanocarriers seem to benefit in penetrating different barriers, but other factors, such as shape, elasticity, and surface charge of the nanoparticles, should also be considered.

Nanoparticle DDS adopts two major strategies to target tumor cells, which are passive targeting and active targeting. Passive targeting takes advantage of the enhanced permeability and retention (EPR) effect which is the property of some tumor cells that readily accumulate macromolecules since the nearby vasculature are leaky and the lymphatic system is malfunctioning. However, not all tumor tissues have a high EPR effect. Active targeting was developed to increase delivery efficiency of low-EPR tumor tissues. In active targeting, specific ligands are attached to the outer layer of the nanoparticles, but this solution has also raised challenging problems. Cellular uptake of macromolecules was not as efficient as the case of small antibodies, and this problem required designing complex nanocarriers to effectively target the tumor cells [52].

To overcome these challenges, researchers are continuously giving a lot of effort. For instance, it is essential to find efficient biomarkers for nanocarrier systems to target the tumor tissue. Moreover, to prevent early release of the loaded drugs, stimuli responsive nanocarrier systems are developed which may interact with the unique macroenvironment around tumor tissues or respond to the external stimuli, like magnetic fields [52]. Undesired interaction with the immune system is usually avoided by PEG coating, but it is also possible to provoke side effects, such as hypersensitivity reactions [53]. The PLGA particle-based DDS breakthroughs of these problems as core–shell microspheres are capable of targeted delivery followed by controlled and sustained drug release. It also adopts a combined therapy strategy to maximize the anti-tumor effect. In this section, we have summarized the recent development of core–shell particles applied in anti-cancer therapy. 

**Figure 3:**
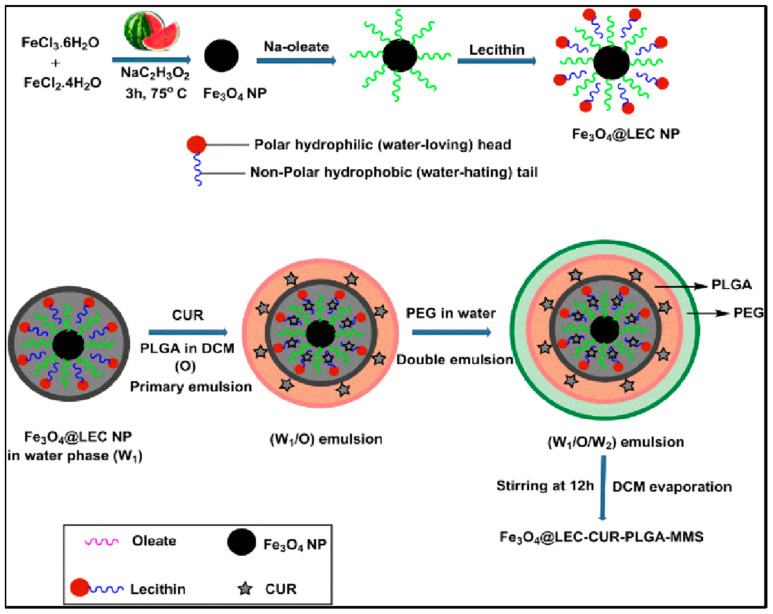
Schematic representation of Fe_3_O_4_@LEC–CUR–PLGA–MMS particle synthesis. Reproduced with permission from [5].

Several successful PLGA-based hybrid microspheres were developed for controlled drug delivery. Materials such as MNPs, gold nanoparticles (NPs), and conducting NPs were utilized as core materials. MNPs can deliver drugs to specific targets under external magnetic fields. Ayyanaar et al. extracted iron oxide nanoparticles (Fe_3_O_4_ NPs) from watermelon rind and used them as a reducing and stabilizing core. Fe_3_O_4_ NPs were incorporated into lecithin (LEC) that is responsive to reactive oxygen species (ROS) for delivery applications, and the antioxidant drug curcumin (CUR) was loaded in the hybrid magnetic system of PLGA microsphere (Fe_3_O_4_ @ LEC–CUR–PLGA–MMS) (Figure 3). Fe3O4 @ LEC–CUR–PLGA–MMS released CUR responding to the ROS environment, especially being cytotoxic against A549 and HeLa S3 cells. The system was toxic to cancer tissues, cytocompatible to normal cells, and could effectively deliver therapeutic agents to disease sites [5]. Another favorable clinical effect was reported by PLGA–Fe_3_O_4_ MNPs carrying quercetin (an anti-cancer agent). This system was introduced by aerosol administration and demonstrated a significant reduction of viable A549 cells [54]. Similarly, the Fang group developed a magnetic responsive PLGA drug-release system loaded with doxorubicin (DOX-MMS). DOX was loaded in the core, and the surface of the microsphere had high contents (28.3wt%) of γ-Fe_2_O_3_ nanoparticles (IOs). After getting exposed to the alternating current magnetic field (ACMF) for 30 min, IOs induced the heat effect (up to 45°C) and triggered shell permeability enhancement of drugs (from 2.8% to 21.6%). In vivo study, the most apoptotic cells were shown (65.4%) in the group with DOX-MMS + ACMF, which highlights the efficiency of chemo–thermal therapy [55]. Photothermal agents play a major role in cancer ablation therapy by converting near-infrared (NIR) light energy to thermal energy. Among other photothermal agents, polypyrrole nanoparticles (PPy NPs) are frequently utilized [56]. Chiang et al. applied PPy NP for localized heat production with simultaneous drug release which can treat bacterial infections efficiently. Hollow microspheres (HMs) with a PLGA shell that has an aqueous core loaded with vancomycin (Van, antibiotics) and PPy NPs are synthesized using the capillary fluidic device. HMs prevented the loss of free drugs at the injection site as they ensured spatial fixation. This system was initiated by external NIR light, and PPy NPs increased local temperature while Van was rapidly released. In vivo study demonstrated a synergistic effect of combining two types of treatments [57]. One more PLGA hollow microsphere system that contains NONOate, a NO donor, and sodium caprate (SC) was developed. The PLGA shell provides protection from hemoglobin (Hb). Under an acidic environment around tumor tissue, NONOate reacts with protons and releases NO bubbles. Layers of SC surfactant then trap and stabilize NO molecules and they are together released from the PLGA shell due to the increased pressure. Outside the PLGA shell, NO molecules slowly diffuse from the encapsulation of SC, providing elongated radiosensitizing effects which inhibit tumor growth [58]. In another study, gold core-mesoporous silica shell rod-like nanoparticles (AuMSSs) and salicylic acid (SA) were loaded inside the microspheres made of PLGA, poly (vinyl alcohol) (PVA), and D-α-tocopherol polyethylene glycol succinate (TPGS). These microspheres were responding to both pH and temperature named NIMPS. Doxorubicin (Dox) loaded AuMSSs and a gas-producing agent (i.e., NaHCO_3_) was added inside the microspheres where gas-generating agents gave the microparticles pH-responding character so that Dox was released under acidic environment. NIMPS under NIR laser irradiation reduced the HeLa spheroids’ size up to 48% [59].

Strategy that combines gene therapy and chemotherapy is optimized when polymeric double-walled microspheres are the carriers because these carriers have predictable releasing pattern and prevent drug/gene from dispersing over time. Agents with low molecular weight and genes are encapsulated in either the inner core or the outer shell. The characteristics of PLGA–core–PLA–shell double-walled microspheres that has Dox and p53 tumor suppressor protein gene (chi-p53) were investigated. Precision particle fabrication method was applied and the resulting average diameter of microspheres was 65–75 μm and uniform thickness was 8–17 μm. The encapsulation efficiency of chi-p53 nanoparticles (25–37%) were not affected by Dox, but the encapsulation efficiency of Dox was reduced from 80% to 32–47% when chi-p53 nanoparticles were present. In vitro study showed the first release of chi-p53 NPs followed by near zero-order releasing chi-p53 and Dox [15]. The clinical performance of these microcapsules on human hepatocellular carcinoma (HepG2) cells showed high expression levels of both tumor suppressor p53 and apoptotic caspase 3 proteins throughout the treatment period. Additionally, it suppressed proliferation and increased apoptosis in HepG2 cells [14].

Paclitaxel (PTX) is one of the most favorable chemotherapeutic agents that effectively interrupts the cell cycle (late G2 phase and M phase) via disrupting the dynamic equilibrium present in the microtubule system [60]. When PTX was applied to epithelial ovarian cancer, it showed significant anti-cancer effects with outstanding penetrating ability up to 80 cell layers [61].

Thus, PTX based chemotherapy has gained attention from several research groups. Recently, Dwivedi et al. prepared polymer–lipid hybrid microparticles loaded with PTX for ovarian cancer treatment. The microparticle was one-step synthesized by co-axial electrohydrodynamic (CEH) method which produced microparticles with uniform size and high encapsulation efficiency (92.17%). The purpose of lipid coating was to advantage the interaction between the PLGA microparticles and tumor cells. In vivo and in vitro study demonstrated that PTX-hybrid-microparticles (PTX-Hyb-MP) have a sustained release profile, increased cellular uptake, and reduced toxicity [62]. Dual drug delivery has more synergistic effect than individual drugs and reduces the probability of developing drug resistance and its subsequent side effects, ultimately enhancing the therapeutic effect [63]. A combinational drug-delivery system of PTX and etoposide (ETP) was designed via loading both of the drugs in PLGA core–shell microspheres by coaxial electrospraying technique to treat osteosarcoma. The resultant PLGA/PTX+ETP (PE) microspheres had an average size of 3.03 μm and encapsulation efficiency of 85.8%. In vitro release profile showed controlled sequential release of two drugs exhibiting a stronger cytotoxic effect on saos-2 osteosarcoma cells compared to treatments using individual drugs [64]. Similarly, dual-drug-loaded multilayer microcapsules can alter the type of drug released at different treatment periods. This is possible when multilayers encapsulate different drugs in an alternating order. During a one-step emulsion solvent evaporation method, the presence of poly ((1,6-bis-carboxyphenoxy) hexane) (PCPH) in the PLGA/PLLA solution can change the distribution of polymer layers. The PCPH layer was formed between the PLLA core and PLGA shell, and the core contained a minor mixture of PLGA and PCPH. Doxorubicin HCL (DOX) (hydrophilic) was loaded inside the PLGA shell while PTX (hydrophobic) was loaded inside the PLLA core. Initial burst of DOX was prevented and PTX releasing was delayed. PLGA/PLLA/PCPH groups also showed reduced spheroid growth rate [49]. A multifunctional cytokine-like recombinant interleukin-2 (rIL-2) also demonstrated a favorable effect on anti-tumor immunity. rIL-2 cytokines were encapsulated inside dextran particles and then encapsulated in PLGA–PLA microspheres to mimic the paracrine depot of cytokine action. Dextran particles protect rIL-2 cytokines’ bioactivity from organic solvents and allows easy encapsulation into PLGA-based microspheres. The release pattern of rIL-2 loaded dextran/PLGA–PLA core–shell microspheres which demonstrated a sustained release for 25 d. In vivo study suggested that the fabricated microspheres have better anti-tumor effect compared to multiple injection of rIL-2 solution possibly due to the longer detention time of microspheres [65].

**Figure 4:**
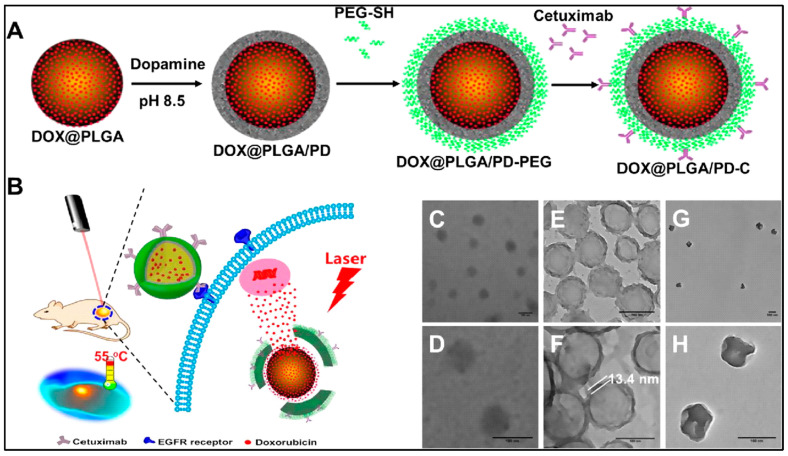
Schematic diagram for the preparation of an epidermal growth factor receptor targeted mussel inspired nanoparticle (**A**), and its proposed mechanism of action (**B**). TEM images of DOX@PLGA (**C**,**D**), DOX@PLGA/PD (**E**,**F**), and DOX@PLGA/PD after NIR irradiation (**G**,**H**). Scale bars are 100 nm in (**C**–**H**). Reproduced with permission from [12].

Along with many combinations of chemotherapy, photothermal therapy, and others, PLGA nanovehicles also allow specific targeting of cancer cells. For example, the core–shell PLGA@PPy NPs drug-delivery system practices both photothermal therapy and chemotherapy for cancer treatment. PLGA encapsulates the curcumin, a potential anti-cancer agent, to overcome the drug’s extreme hydrophobicity. The curcumin loading content and encapsulation efficiency was 6.3% and 67.6% each. The PLGA core is then used to grow PPy on its surface to allow photothermal ablation of cancer cells. The synergistic effect of combining photothermal therapy and chemotherapy resulted in intensive cytotoxicity. Exposing to 808 nm NIR laser at 1.2W cm^−2^ for 5 mins was enough to kill 90% of the cells treated by the PLGA(Cur)@PPy (Cur concentration was 10 μg/mL) [11]. Similarly, mussel-inspired PLGA/polydopamine core–shell nanoparticles loaded with doxorubicin were designed for head and neck cancer photothermal and chemotherapy. These nanoparticles can effectively enter cancer cells using its epidermal growth factor receptor (EGFR) antibody, since cancer cells have overexpressed EGFR. Like mussels, polydopamine (PD) can adhere to the surface of biomolecules containing thiol or amine. PD also has the ability to absorb NIR light and generate heat (Figure 4). Using PLGA–PD core–shell nanoparticles solves the problem of most photothermal converting agents being not biodegradable, and as their heat generation is dependent on nanoparticle concentration, the overheating issue can also be prevented [12].

**Figure 5:**
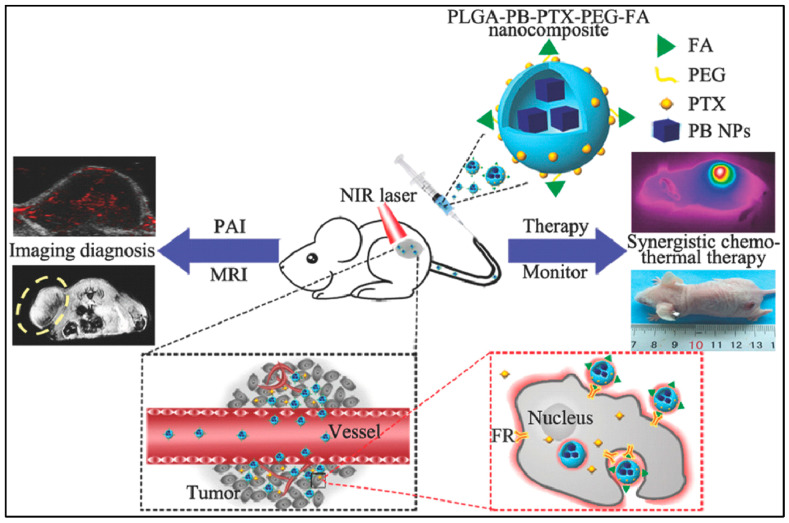
Schematic representation of PLGA–PB–PTX–PEG–FA nanococktail and its corresponding targeted theranostic performance, including PA/MRI-guided synergistic NIR-triggered PTT and chemotherapy. Reproduced with permission from [13].

For breast-cancer treatment, PLGA–Prussian blue nanoparticles (PB NPs)–PTX–PEG–folic acid (FA) ‘nanococktails’ were fabricated (Figure 5). PTX is a cytotoxic agent for chemotherapy, and PB NPs have excellent photothermal conversion ability for photothermal treatment. FA is used for targeting cancer cells. Especially, PLGA–PB–PTX–PEG–FA nanococktails can play a role as a contrast agent for photoacoustic (PA)/magnetic resonance imaging (MIR) dual-mode diagnostic imaging which is demonstrated in both in vivo and in vitro experiments [13].

**Figure 6:**
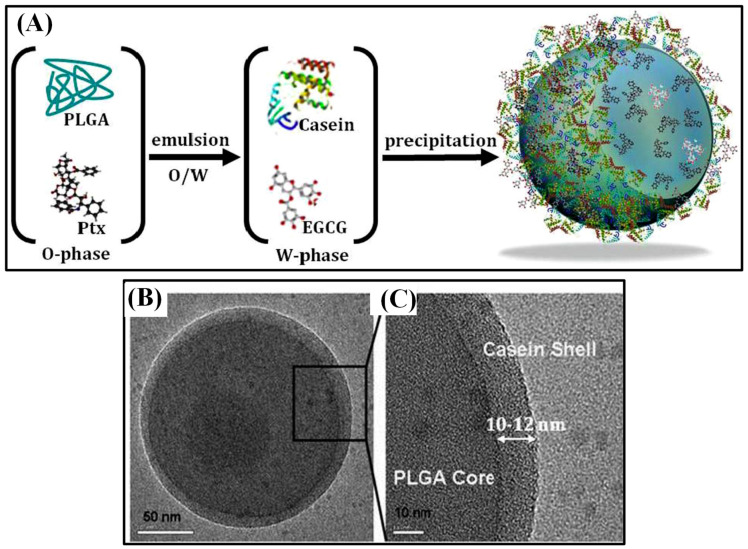
Schematic illustration of dual drug-loaded PLGA–casein core/shell nanoparticles fabrication (**A**). TEM image of core/shell nanoparticles (**B**) and high-magnification TEM (**C**). Reproduced with permission from [68].

Few other chemotherapeutic agents showed anti-cancer activity. For instance, Cisplatin (CDDP) is a chemotherapeutic agent that triggers cell apoptosis by causing significant DNA damage. Targeted delivery of such a cytotoxic drug is necessary to increase its clinical efficiency. CDDP-loaded PLGA nanoparticles were fabricated via electrohydrodynamic atomization (EHDA) method. The resulting core–shell (CS) structured nanoparticles showed the most sustained release of CDDP after a short initial burst. Cytotoxicity of the nanoparticles were like that of free CDDP, yet the activation site was internalized into the endolysosomal compartments of cancer cells [66]. Delivered of another chemotherapy drug Gemcitabine via conventional hydrophobic carrier matrices is inefficient as it doesn’t carry charged groups. Since tumor cells ingest more albumin than normal cells, gemcitabine was cross-linked with human serum albumin (HSA) inside the hydrophilic space of CSNPs and the core was coated by a PLGA shell. HSA–core PLGA–shell nanoparticles showed core–shell structure and had an optimized size of 241 ± 36.2 nm and an encapsulation efficiency of 41.52%. In vitro and in vivo release study showed a sustained release rate (in vitro, 50% was released within an hour and 96.56% released after 11h, compared by 95.73% released in the aqueous solution in 1 h) due to the solidified albumin core and hydrophobic PLGA shell. This delayed diffusion of the drug would lower its toxicity and prevent being degraded by enzymes [67]. DDS becomes even more efficient if multiple drugs can be sequentially released at a desired timing with a sustained release pattern. A chemically distinct (hydrophobic/hydrophilic) model drug has been entrapped and sequentially released by compartmentalized nano vehicles. Hybrid core/shell system of PLGA–casein nanocarrier (190 nm in size) was designed as a polymer-protein-based nanosystem (Figure 6). Hydrophobic PTX was loaded in the hydrophobic PLGA core by emulsion, and hydrophilic epigallocatechin gallate (EGCG) was loaded inside the casein shell by precipitation. The encapsulation efficiency of PTX was 95.7%, and the EGCG was 76.8%. EGCG was released first and PTX was released later, which lasted for 7 and 12 d, respectively. Nanoencapsulated PTX and EGCG improved circulation half-life, plasma concentration, and residence time in comparison to the bare drugs. PLGA–casein nanocarriers were hemo-, cyto-, and immunocompatible when their concentration was 1mg/mL [68]. The co-delivery of EGCG and PTX improved the anti-tumor effects by balancing the cytotoxic and cytostatic properties. Additionally, the activation of NF-κB pathway by PTX was balanced by anti-NF-κB potential of EGCG and improved its cytotoxic effect significantly. Using the surface receptor, nanoparticles were targeted to breast-cancer cells [69].

## 3. Tissue Regeneration

Tissue regeneration aims to restore tissues damaged by injury or disease. Autologous cell therapy is conventionally used, in which host cells are isolated, proliferated, and injected back into the host. This method was preferred since it can avoid complicated problems of allogeneic transplantation. However, autologous cell therapy still has issues because cells that failed to adhere easily die. As a solution to this limitation, tissue regeneration using microparticles should develop a scaffold to support and hold cells as well as deliver drugs to proliferate and differentiate cells [70]. Furthermore, the PLGA core–shell delivery system has been proved to be promising for biomedical applications, like tissue engineering [27].

### 3.1. Bone Regeneration

The bone-healing process follows sequential order of chemotaxis, cell proliferation, matrix synthesis, and differentiation [71]. Controlled and sequential release of drugs which is facilitated by microspheres effectively serves this purpose. Biodegradable materials are selected for bone-tissue engineering, and thus the removal process of scaffolds are not required after the defect is healed [72]. Bone morphogenetic protein (BMP2) are frequently incorporated into scaffolds and exhibit effective bone regeneration [73].

Recombinant human bone morphogenetic protein-2 (rhBMP-2) loading PLGA core–shell microspheres were synthesized via coaxial electrospraying, which enhanced cell proliferation and facilitated bone regeneration. Coaxial electrospraying allowed manufacturing of small sized particles with the range from 2.5 to 8 μm. Bovine serum albumin (BSA) stabilized and protected rhBMP-2’s bioactivity. Moreover, the microspheres showed an initial burst followed by a stable release rate [72]. In another study, the PLLA–PLGA core–shell double-walled microsphere achieved the different release patterns of FGF-2 and BMP-2. Releasing these growth factors simultaneously and continuously for 4 w resulted in the resorption of grafted bone in vivo. Additionally, it enhanced osteoclastogenesis in vitro compared to single GF treatments [74]. Another core–shell microsphere increased osteogenesis and angiogenesis in bone marrow mesenchymal stem cells and microvascular epithelial cells respectively in vitro with the encapsulation of BMP-2 in PLGA core and VEGF in PDLLA shell. The sequential release of BMP2 and VEGF enhanced the formation of new bone in critical-sized calvaria bone defect. VEGF encapsulated inside the PDLLA shell was released during the first 10 d. On the other hand, core-loaded BMP2 showed a relatively sustained release pattern over time [75]. Releasing osteogenic biomolecules jointly at different doses facilitated the osteogenic gene. The osteogenic biomolecules of BMP-2 and dexamethasone from PLGA–alginate core–shell microcapsules upregulated the osteogenic gene level, including osteopontin, type I collagen, and osteocalcin [76].

**Figure 7:**
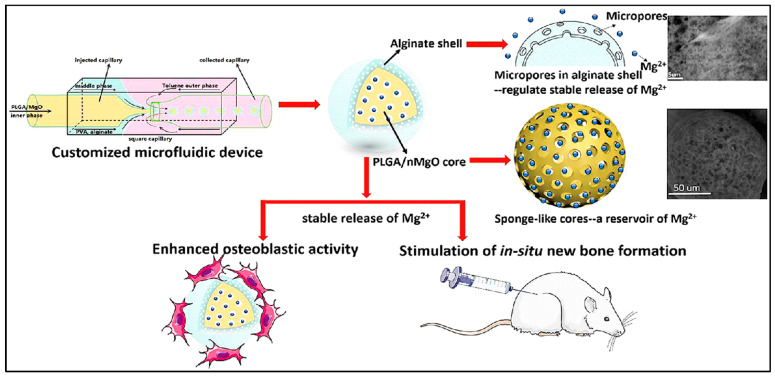
Schematic representation of PLGA/magnesium oxide nanoparticle core with alginate shell microsphere prepared using microfluidic capillary device and their osteoblastic activity in vitro and in situ bone regeneration in vivo. Reproduced with permission from [78].

Magnesium ion (Mg^2+^), the most abundant element in bone tissue, significantly affects the biomechanics and mineral density of natural bone. The sustained release of Mg^2+^ significantly facilitated bone defect regeneration [77]. Precisely controlling the Mg^2+^ release can successfully activate in situ bone regeneration. Using the customized microfluidic capillary device, a sponge-like monodisperse PLGA/magnesium oxide nanoparticle core with alginate shell delivery system was designed (Figure 7). Alginate hydrogel plays a role in reducing the outflow of Mg^2+^ at ~50 ppm accurately for 2 w in vitro, in which the concentration of Mg^2+^ of 50–200 ppm effectively promotes the proliferation and differentiation of pre-osteoblasts. Young’s modulus of regenerated bone tissue of PLGA/MgO-alginate microspheres have been restored ~96% of the damaged tissue while the PLGA and PLGA/MgO groups induced the bone moduli 65% and 71%, respectively [78].

### 3.2. Cartilage Regeneration

Cartilage is a supporting connective tissue that exists throughout our body, especially on the surfaces of our joint bones. Cartilage tissue lacks enough self-regenerative ability to restore damage since it has sparse cellularity, little extracellular matrix, and lacks blood vessels and the nerve supply [79]. Treatments such as autologous implantation and stem cell therapy exhibited successful repairs. However, these therapies are not applicable when stem cells or autologous chondrocytes are not available [8]. Tissue engineering using biocompatible hydrogels combined with drug-loading microspheres can provide a solution.

**Figure 8:**
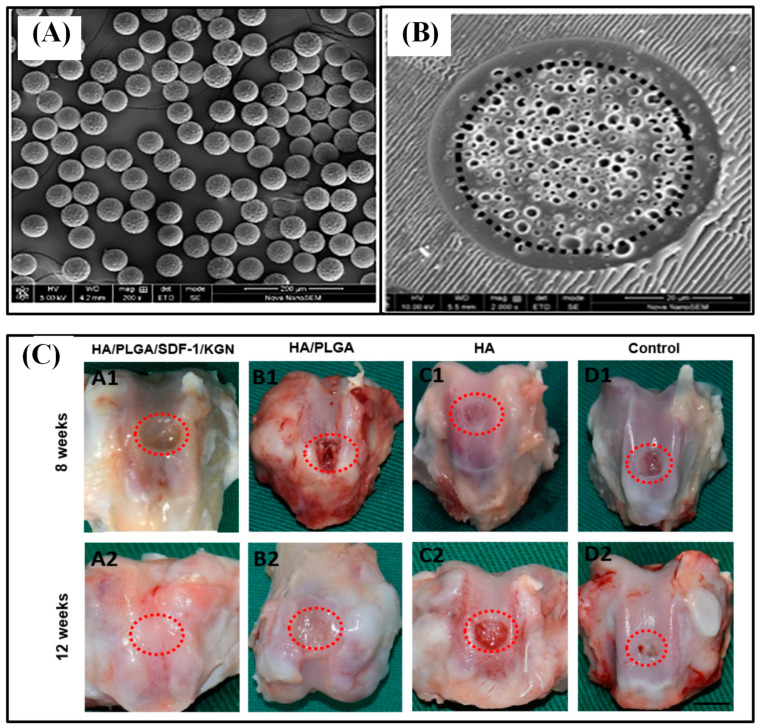
SEM image of PLGA core–shell microspheres (**A**) and its cross-sectional morphology (**B**). Gross evaluation of the repaired tissue at 8 and 12 w (**C**). The scale bar is 3.5 mm. Reproduced with permission from [8].

Thermosensitive injectable hydrogel called poly (N-isopropylacrylamide) (PNIPAM) was blended with hyaluronic acid (HA). Then, chitosan-g-acrylic acid coated PLGA (ACH-PLGA) particles were incorporated into the PNIPAM/HA hydrogel, which enhanced cartilage tissue regeneration. The role of HA was reducing the synthesis of PNIPAM gel up to 50% and increasing the bioactive property of the hydrogel. To enhance the mechanical features of the system, ACH-PLGA microparticles were incorporated so that the allylic group on the chitosan-g-acrylic acid shell promotes the cross-linking ability of PNIPAM. Melatonin was used as a model drug, and the novel injectable hydrogel demonstrated enhanced mechanical properties, reduced syneresis, sustained release, and high bioactive property [80]. The co-delivery of a chemokine (stromal cell-derived factor-1, SDF-1) and a chondroinductive molecule (kartogenin, KGN) via microspheres incorporated in hyaluronic acid (HA) scaffold also showed the ability of articular cartilage defect repair (Figure 8). PLGA microspheres were fabricated by using a microfluidic technology, loading SDF-1 in the core and KGN in the shell. It concluded that the shell-loaded KGN (EE of 96.08%) would release faster during the first month while the core-loaded SDF-1 (EE of 97.04%) would match the release rate around 30 d to 50 d. PLGA microspheres were incorporated into HA cross-linked scaffold to provide an advantageous microenvironment for cartilage regeneration as well as to hold both microspheres and home cells. In vitro study of the HA/PLGA/SDF-1/KGN group showed excellent tissue regeneration, the engineered tissue well integrated with normal ones while exhibiting normal structure, having an even surface and thickness, and being more mature [8]. DS, a member of the glycosaminoglycans family, plays a role in activating adhesion molecules and facilitating cell proliferation. Alginate/dermatan sulfate (Alg/DS) or alginate/chitosan/dermatan sulfate (Alg/CS/DS) was loaded inside mPEG-PLGA microcarriers by applying coaxial ultrasonic atomization technique. The optimal condition for encapsulating drugs in core–shell structured microparticles was achieved by adjusting the optimal viscosity and the flow rate ratio of the polymer. The Alg/CS/DS group demonstrated a better sustained release profile and increased fibroblast proliferation than Alg/DS particles. This is due to the formation of the polyelectrolyte complexes between CS (positively charged) and DS (negatively charged). mPEG-PLGA allowed further prolonged drug release while prohibiting initial burst of a drug and also allowed cells to adhere to its surface [70].

### 3.3. Periodontal Regeneration

Previous periodontal tissue or dentoalveolar structure damage treatment includes exogenous growth factors, grafts that replaced bone, guided tissue regeneration (GTR), and tissue engineering methods. Clinical outcomes vary to a high degree, and it is assumed that precisely controlling the drug-release rate is required because dentoalveolar regeneration is driven by a cascade of regulated events [81]. Core–shell nanoparticles allow both controlled and sequential delivery of drugs to execute it. 

Chang et al. achieved the simultaneous release of platelet-derived growth factor (PDGF) and simvastatin using poly-D,L-lactide (PDLLA) core (PLGA)–shell microsphere. PDGF is a hydrophilic molecule that mediates mitogenesis, and simvastatin is a hydrophobic differentiation factor that assists proliferation and reduces apoptosis and inflammation of cells. Co-axial electrohydrodynamic atomization (CEHDA) method allows generating a double-walled core–shell structure of uniform size. Using CEHDA, PDGF-simvastatin double-walled PLGA (PDLLA) microparticles of 10–20 μm were fabricated. PDLLA was selected as a core material since it should slowly degrade so that the agent is not prematurely released. The encapsulation efficiency (EE) of simvastatin core–PDGF shell structure (SP) was 82.5% and 51.4% each, while the EE of PDGF core–simvastatin shell (PS) was 92.2% and 71.3 each. In vitro study revealed that SP microspheres have a sequential release pattern with initial burst of PDGF while PS microspheres have a parallel release pattern of both agents. In vivo study demonstrated that simultaneously releasing PDGF and simvastatin from PS microparticles could decrease cell death as simvastatin downregulates the inflammatory cytokine production and PDGF upregulates the pathways that reduced apoptosis [18]. Sequentially releasing PDGF (as a mitogen) and simvastatin (as a differentiation factor) can decrease heat-induced osteonecrosis and facilitate osteogenesis of alveolar bone defects. Moreover, co-delivery of PDGF and simvastatin can regenerate functionally oriented periodontal ligament (PDL) and cementum. The microsphere generated from CEHDA technique indicated that sequentially delivering PDGF-simvastatin could facilitate cementogenesis and PDL re-alignment as well as osteogenesis and bone maturation [20]. Rapidly releasing PDGF enhanced early mitogenesis while slowly releasing simvastatin increased osteogenic differentiation [82]. The research group compared the performance of five different PDLLA–PLGA microspheres: encapsulating bovine serum albumin (BB), PDGF alone (XP), simvastatin alone (SB), PDGF-core–simvastatin-shell (PS), and simvastatin-core–PDGF-shell (SP) for the sequential release of (PDGF) and simvastatin. The results concluded that SP groups which demonstrated initial burst of PDGF, and relatively slow release of simvastatin significantly promoted osteogenesis. SP groups were better at increasing the osteoclastic cells than PS groups since continuous release of PDGF from the core tended to interfere with simvastatin’s dentoalveolar osteogenesis [19].

**Figure 9:**
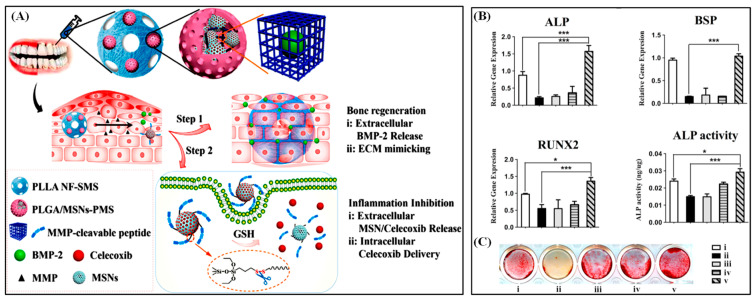
Schematic representation of PLGA/MSNs-PMS immobilized PLLA spongy nanofibrous micro-scaffold and dual responsive bio-molecules delivery designed complex (**A**). The gene expression of bone-related marker ALP, BSP, and RUNX2 (**B**) and the ALP activity (**C**) of inflamed bone marrow mesenchymal stem cell cells after treatment by different formulations. Reproduced with permission from [21].

To treat periodontal diseases in a way that (1) is selective to locations, (2) automatically adjusts drug dosage, and (3) has both bone regenerating and anti-inflammatory effect. Poly (lactic-co-glycolic acid)/mesoporous silica nanocarriers core–shell porous microsphere (PLGA/MSNs-PMS) encapsulated poly (L-lactic acid) PLLA spongy nanofibrous micro-scaffold was synthesized. This system is triggered by over-expressed matrix metalloproteinases (MMP) in ECM. Since MMPs’ expression level is proportional to the inflammation level, the release amount is dependent on the severity of inflammation. BMP-2, a bone growth factor, is first released in the extracellular matrix from PLLA porous microspheres (Figure 9). PLLA microspheres are advantageous for extracellular release since it has sufficient loading spaces for large molecules and does not go under endocytosis. Along with BMP-2, PLGA/MSNs-PMS is released from the PLLA microsphere since MSNs-PMS core is MMP-cleavable. MSNs are selected since their surface is readily functionalized so that, for example, they can react to pH, light, GSH, ROS, or enzyme stimuli. Escaped celecoxib (anti-inflammatory drug)/MSNs undergoes endocytosis, cleaved by GSH, and releases celecoxib in the intracellular space. In this way, BMP-2 promotes bone regeneration in extracellular space while celecoxib reduces inflammation in intracellular space [21]. Moreover, we have summarized the core–shell particles for biomedical applications, including their preparation method in Table 1.

## 4. Conclusion and Future Perspectives

PLGA core–shell microspheres are widely developed as an alternative drug-delivery system (DDS). These microspheres allow drugs to remain bioactive, controls release kinetics, localizes treatment area, and it can be modified to respond to various environmental stimuli such as light. PLGA has excellent biocompatibility and biodegradability, but its highly hydrophobic nature limits its ability to encapsulate hydrophilic drugs. The design of core–shell structures overcome this issue by improving the encapsulation efficiency and release kinetics. Core–shell allows for a sequential delivery of multiple drugs that are loaded in the core or shell compartments. These multiple factors loaded core–shell particles are used for various biomedical applications. However, clearance of these particles from the body, and interactions of these particles with particular cells are the major challenges to be considered for the clinical applications. Therefore, the design of a hydrophilic shell, like PEG, may improve the clearance [43]. In addition, the surface modification of the particle by providing a binding site will improve the chances of interaction between the particle and target cells [41]. The recent development of stimuli-responsive PLGA core–shell particles may be a new approach for controlling drug release depending on the microenvironment condition [83]. Furthermore, considering a hydrophilic shell such as PEG, chitosan, or alginate shell, can control initial burst while prolonging the drug release [38,39,43]. Finally, commercialization is challenging for these particles because the conditions required for synthesis are generally hard to achieve, thus its reproducibility and scaling up is questioned.

## Figures and Tables

**Table 1:** Summarizes the PLGA core–shell micro/nano particles for various biomedical applications.

Material		Fabrication Technique	Bioactive Molecule	Application	Ref.
Core	Shell				
Aqueous core	PLGA	Modified internal phase separation	Risedronate sodium (hydrophilic)	Osteoporosis	[34]
PLGA or PLA	PNIPAM	Single emulsion + aqueous free radical precipitation polymerization	Ramipril	Anti-hypertensive	[47]
PLGA	PEG	Nanoprecipitation + self-assembly	Docetaxel	Chemotherapy	[43]
PLGA	GC	W/O/W emulsion–solvent evaporation	bFGF/CHA	Chronic wound	[37]
QRuNPs	PLGA–DS	Double emulsion	Resveratrol	Rheumatoid arthritis	[41]
BSA	PLGA	W/O/W double emulsion	Gemcitabine	Anti-cancer Agent	[44]
PLGA	PLGA	W/O/W double emulsion	INH	Mycobacterium tuberculosis	[42]
Ca Alg	PLGA	W/O/W double emulsion	MCA (hydrophilic)	Anti-emetic and gastroprokinetic agent	[32]
PLGA	Ppy NPs	Quasi-emulsion diffusion + SPG membrane emulsification	Curcumin (hydrophobic, poor bioavailability)	Anti-cancer	[11]
PLGA	Casein	O/W emulsion precipitation	PTX (hydrophobic) EGCG (hydrophilic)	Anti-cancer	[68]
PLGA	PLGA	Single emulsion using vegetable oil	Rhodamine B	Hydrophilic model drug	[6]
PLGA	PLGA	W/O/W double emulsion	Goserelin acetate	Prostate cancer hormonal therapy	[33]
PVP or PLGA	PEG	Emulsion solvent evaporation	Amphotericin B	Fungal infections	[45]
PLA or PLGA	Pluronic F127	Nanoprecipitation	(±)-α-Tocopherol	Vitamin E	[46]
HSA	PLGA	Modified W/O/W double emulsification solvent evaporation	Gemcitabine (hydrophilic)	Anti-cancer agent	[67]
PLGA	Alginate	Capillary microfluidic method	Rifampicin	Antibiotic	[39]
PLGA	PLGA	Dual capillary electrospray	Budesonide (hydrophobic) Theophylline (hydrophilic)	Asthma	[10]
LP powder, water, gelatin, or F127	PLGA	Modified w/o/w double emulsification solvent evaporation	LP (hydrophilic, small)		[30]
Fe3O4 NPs	PLGA/PEG	W/O/W double emulsion	Lecithin curcumin	Reactive oxygen species responsive antioxidant	[5]
Aqueous core	PLGA	Capillary fluidic device	Vancomycin, PPy NPs	Photothermal agent	[57]
PLGA	Lipid	CEH process	PTX	Ovarian cancer	[62]
PLGA	PD	Modified emulsion	DOX/ anti-EGFR antibody	Head and neck cancer photothermal and chemotherapy	[12]
PLLA	PLGA	One-step emulsion solvent evaporation	PTX (hydrophobic) DOX HCl (hydrophilic)	Anti-cancer agent	[49]
AuMSSs	PLGA	W/O/W double emulsion	DOX/ salicylic acid	pH- and thermo- responsive cervical cancer treatment	[59]
PLGA	Casein	Emulsion–precipitation	PTX/EGCG	Targeted breast-cancer therapy	[69]
Cisplatin	PLGA	EHDA	CDDP	Cancer chemotherapy	[66]
PB NPs	PLGA/PEG	Modified W/O/W double emulsification solvent evaporation	PTX/FA	Breast-cancer targeted photothermal and chemotherapy	[13]
PLGA	PLLA	Precision particle fabrication	DOX/chi-p53	Hepatocellular carcinoma chemotherapy and gene therapy	[14]
PLGA	PLA	Precision particle fabrication	DOX/chi-p53	Combined gene therapy and chemotherapy	[15]
PLGA	PLGA	Coaxial electrospraying	PTX + ETP	Osteosarcoma	[64]
PLGA	PLA	Stabilizing aqueous–aqueous emulsion	rIL-2	Anti-tumor agent	[65]
PLGA	PLA	W/O/W double emulsion	BMP2 17β-estradiol	Osteoporosis bone regeneration	[17]
PLGA	Alg	Customized microfluidic capillary device	Mg^2+^	In situ bone regeneration	[78]
PLGA	PLGA	Coaxial electrospraying	rhBMP-2	Bone regeneration	[72]
PLGA	CH-g-AA	Single emulsion solvent evaporation + physical adsorption	Melatonin	Cartilage tissue regeneration	[80]
mPEG	PLGA	Coaxial ultrasonic atomization	Alg/CS/DS	Dermatan sulfate deliver system	[70]
PLGA	PLGA	W/O/W microfluidic emulsion	SDF-1 KGN	Cartilage tissue regeneration	[8]
PDLLA	PLGA	CEHDA	PDGF/simvastatin	Dentoalveolar Regeneration	[19]
PDLLA	PLGA	CEHDA	PDGF/simvastatin	Periodontal tissue regeneration	[18]
PDLLA	PLGA	CEHDA	PDGF/simvastatin	Periodontal tissue regeneration	[20]
MSNs	PLGA	Modified w/o/w double emulsification	Celecoxib BMP-2	Periodontal disease	[21]

**Abbreviations:** Alg: alginate; ACH–PLGA: Chitosan-g-acrylic acid coated PLGA; AuMSSs: Gold core-mesoporous silica shell rod-like nanoparticles; CH-g-AA: Chitosan-graft-acrylic acid; GC: Glycol chitosan; mPEG: Methyl–PEG; MSNs: Mesoporous silica nanocarriers; PB NPs: Prussian blue nanoparticles; PCPH: Poly[(1,6-bis-carboxyphenoxy) hexane; PEG: Poly (ethylene glycol); PD: Polydopamine; PDLLA: Poly (D,L-lactide); PHBV: Poly(3-hydroxybutyrate-co-3-hydroxyvalerate); PLA: Poly (lactic acid); PLGA: Poly (lactic-*co*-glycolic acid); PLGA–DS: PLGA–dextran sulfate; PLLA: Poly (L-lactic acid); PNIPAM: Poly (N-isopropylacrylamide); PPy NPs: Polypyrrole nanoparticles; PVP: Polyvinyl pyrrolidone; QRuNPs: Quadrilateral ruthenium nanoparticles; bFGF: Basic fibroblast growth factor; BMP-2: Bone morphogenetic protein -2; CDDP: Cisplatin; CHA: Chlorohexidine acetate; chi-p53: Chitosan–DNA nanoparticles containing the gene encoding the p53 tumor suppressor protein; CS: Chitosan; DOX: Doxorubicin; DS: Dermatan sulfate; EGCG: Epigallocatechin gallate; EGFR: Epidermal growth factor receptor; ETP: Etoposide; FA: Folic acid; HA: Hyaluronic acid; HGF: Hepatocyte growth factor; HP- γ-CD: 2-hidroxipropil-γ-ciclodextrin; HAS: Human serum albumin; INH: Isoniazid (Pyridine 4-carboxylic acid hydrazide); KGN: Kartogenin; LP: losartan potassium; MCA: Metoclopramide hydrochloride; Mg^2+^: Magnesium ion; Nano-HAP: Hydroxyapatite nanoparticles; PDGF: Platelet-derived growth factor; PPy NPs: Polypyrrole nanoparticles; PTX: Paclitaxel; rIL-2: Recombinant interleukin-2; rhBMP-2: Recombinant human bone morphogenetic protein-2; SDF-1: Stromal cell-derived factor-1; CEH: Coaxial electrohydrodynamic; CEHDA: Coaxial electrohydrodynamic atomization; and EHDA: Electrohydrodynamic atomization.

## Data Availability

Not applicable.
